# Reply: Childhood leukaemia and population movements in France, 1990–2003

**DOI:** 10.1038/sj.bjc.6604552

**Published:** 2008-09-30

**Authors:** S Bellec, J Clavel

**Affiliations:** 1 INSERM U754, Hôpital Paul Brousse, F-94807 Villejuif, France; 2 Université Paris XI, F-94276 Kremlin-Bicêtre, France; 3 French Registry of Childhood Haematopoietic Malignancies, F-94807 Villejuif, France

**Sir,**

We thank Professor Wakeford for his comments on our recent paper, which evidenced an increased risk of childhood acute leukaemia in isolated French communes that were subject to highest inter-census population movements, more markedly above a given threshold of population density (Bellec *et al*, 2008).

Population variations in France during the inter-census period (1990–1999) were so skewed on the commune scale that we decided to consider the proportion of individuals who changed address between 1990 and 1999 as a proxy measure of population movements. We agree that a high proportion of in-migrants could be compatible with a population increase, but not necessarily. This response allows us to describe the relation between population changes and the proportion of in-migrants, which was not evoked in our paper.

The relative variation of population size was significantly associated with the proportion of in-migrants in isolated communes. The higher the proportions of in-migrants from distant communes, the higher were population changes between 1990 and 1999. Isolated communes with less than 8% of in-migrants were thus associated with a 2.3% population increase on average, whereas those with the highest proportions of in-migrants (⩾14%) saw their population increase of 4.1% on average. However, in contrast to population changes mentioned in other studies, particularly Kinlen's, such an increase could not be regarded as a marked increase.

Our study highlights not only the overall involvement of in-migration but also the major role of the migration distance. Although no association was evidenced with the overall proportion of in-migrants, a marked increase risk of childhood leukaemia was found in communes of highest average migration distances, particularly in isolated communes. This result may reflect the fact that the further the migrants came, the less likely it was that they shared with the local population a common immune status with respect to the viral agent hypothetically involved in the aetiology of childhood leukaemia.
